# Emerging roles of mitochondria in animal regeneration

**DOI:** 10.1186/s13619-023-00158-7

**Published:** 2023-05-05

**Authors:** Yun Zhao, Chong Gao, Xue Pan, Kai Lei

**Affiliations:** 1 Westlake Laboratory of Life Sciences and Biomedicine, Key Laboratory of Growth Regulation and Translational Research of Zhejiang Province, School of Life Sciences, Westlake University, Hangzhou, Zhejiang 310024 China; 2 Institute of Biology, Westlake Institute for Advanced Study, Hangzhou, Zhejiang 310024 China; 3 Fudan University, Shanghai, China; 4 Key Laboratory of Novel Targets and Drug Study for Neural Repair of Zhejiang Province, School of Medicine, Hangzhou City University, Hangzhou, China; 5 College of Life Sciences, Zhejiang University, Hangzhou, Zhejiang China

**Keywords:** Mitochondria, Regeneration, Planaria, Aging, Model organisms

## Abstract

The regeneration capacity after an injury is critical to the survival of living organisms. In animals, regeneration ability can be classified into five primary types: cellular, tissue, organ, structure, and whole-body regeneration. Multiple organelles and signaling pathways are involved in the processes of initiation, progression, and completion of regeneration. Mitochondria, as intracellular signaling platforms of pleiotropic functions in animals, have recently gained attention in animal regeneration. However, most studies to date have focused on cellular and tissue regeneration. A mechanistic understanding of the mitochondrial role in large-scale regeneration is unclear. Here, we reviewed findings related to mitochondrial involvement in animal regeneration. We outlined the evidence of mitochondrial dynamics across different animal models. Moreover, we emphasized the impact of defects and perturbation in mitochondria resulting in regeneration failure. Ultimately, we discussed the regulation of aging by mitochondria in animal regeneration and recommended this for future study. We hope this review will serve as a means to advocate for more mechanistic studies of mitochondria related to animal regeneration on different scales.

## Background

Mitochondria are highly specialized and dynamic double-membrane bound organelles in eukaryotic cells. As the powerhouses of the cell, mitochondria enable the eukaryotes to efficiently generate ATP from energy-rich molecules (Chan 2006). Over an evolutionary timescale, mitochondria have been derived from alphaproteobacteria, evolving to exist in symbiosis with eukaryotic cells. Mitochondria retain genetic material from this endosymbiotic event in mitochondrial DNA (mtDNA) that only encodes for 13 subunits of the electron transport chain as well as 2 rRNAs and 22 tRNAs (Gray 2012). Through studies over the last fifty years, mitochondria have been realized as signaling centers for multiple fundamental processes in addition to being a “powerhouse”. Anterograde and retrograde communication between mitochondria and the nucleus are essential in maintaining mitochondrial function and overall cell health (Chandel 2015; Tan and Finkel 2020). Proteins required for mitochondrial biogenesis are translated from the nuclear genome. Retrograde signaling from mitochondria to the nucleus, often in the form of metabolites, translocated mitochondrial proteins, and mtROS all consequently regulate several cellular processes including cell cycling, apoptosis, immune response, and epigenetic regulation (Vyas et al. 2016; Wallace 2012; Weinberg et al. 2015). Dysregulation of mitochondrial-nuclear communication has been demonstrated to play a direct role in aging and several pathologies (Liesa et al. 2009; Murphy and Hartley 2018; Nunnari and Suomalainen 2012). Since multiple fundamental functions have been extensively reviewed in other places, we focus instead on the related aspects of animal regeneration in this review.

Mitochondrial networks display dynamic morphologies under various physiological and pathological conditions (Buck et al. 2016; Chan 2020; Iwata et al. 2020; Khacho et al. 2016). These mitochondrial networks provide a system of interplay with other cellular organelles, such as the endoplasmic reticulum (ER), lysosomes, cytoskeleton, and nucleus (Szymanski et al. 2017; Wong et al. 2019). Many fundamental functions of mitochondria have been examined and understood in the process of animal regeneration, which will be comprehensively summarized in this review. However, most studies on molecular mechanisms have heavily relied on relatively simpler models such as cell and internal tissues and organs. It is unclear whether the regulation is the same or whether additional mechanisms exist in more complicated environments, such as structure and whole-body regeneration. It also remains to address whether and how mitochondria regulate distinct transcriptional programs in different cell types and tissue environments such as aging during animal regeneration.

Calcium ions (Ca^2+^) act as secondary messengers, with calcium balance being regulated by interactions among mitochondria, ER, and lysosomes (Berridge et al. 2000; Cardenas et al. 2010; Hirabayashi et al. 2017; Raffaello et al. 2016). Various cellular stimuli can induce the transcription-independent increase of Ca^2+^, which triggers a morphological change of mitochondrial networks and increases the mitochondrial membrane potential and reactive oxygen species (ROS) production (Giacomello et al. 2020; Tan and Finkel 2020). Maintenance of proper mitochondrial networks is crucial in animal development, homeostasis, and stem cell self-renewal and fate (Hong et al. 2022; Iwata et al. 2020; Khacho et al. 2016; Mansell et al. 2021; Pei et al. 2018; Prieto et al. 2020; Zhong et al. 2019). Because mitochondrial network morphology is coupled with metabolic activities such as oxidative phosphorylation (OXPHOS) and glycolysis, the downstream mechanisms across different regeneration contexts are worth reviewing.

Mitochondria-derived damage-associated molecular patterns (mitoDAMPs) can be released from dying cells in injured tissues. These mitoDAMPs can serve as initiation factors for an early immune response to injuries (Krysko et al. 2011; Oka et al. 2012; Westhaver et al. 2022; Zhang et al. 2010). Moreover, fully functional mitochondria can be released directly or in extracellular vesicles for uptake by recipient cells (Al Amir Dache et al. 2020; Boudreau et al. 2014; Chiu et al. 2003; Jiang et al. 2016; Kitani et al. 2014; Maeda and Fadeel 2014; Pollara et al. 2018; Torralba et al. 2016). Indeed, the intercellular mitochondrial transfer has been implicated in physiological homeostasis and diseases, occurring in macrophages in adipose tissues, ischemic stroke, and cancers (Chang et al. 2019; Dong et al. 2017; Griessinger et al. 2017; Hayakawa et al. 2016; Pollara et al. 2018; van der Vlist et al. 2022; Zhu et al. 2016). The necessity of the released or transferred mitochondria, as well as their precise role in animal regeneration, are interesting areas of study.

## Mitochondrial response in animal regeneration across different model organisms

**Fig. 1:**
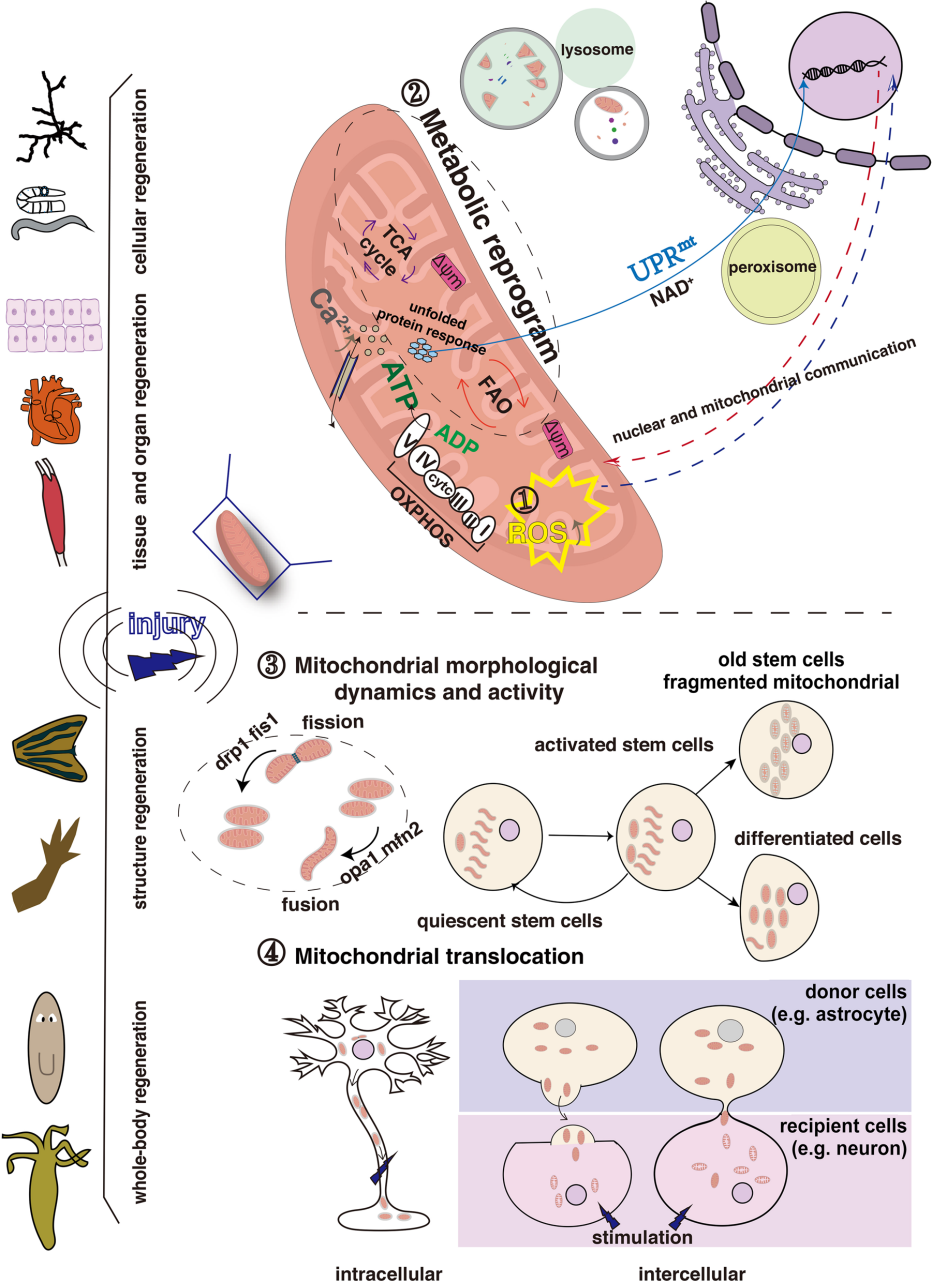
Schematic summarizing mitochondrial function in multiple types of regeneration including cellular, tissue, organ, structure, and whole-body regeneration. Throughout the regeneration process, mitochondria are involved in several pathways including ROS, metabolic reprogramming, mitochondrial morphological dynamics and activity, mitochondrial translocation, and transcriptional regulation. Reactive oxygen species (ROS), cytochrome c (cyt c), oxidative phosphorylation (OXPHOS), adenosine triphosphate (ATP), tricarboxylic acid cycle (TCA), fatty acid oxidation (FAO), mitochondrial membrane potential (Δψm), mitochondrial unfolded protein response (UPR^mt^), nicotinamide adenine dinucleotide (NAD^+^)

Animal regeneration can be categorized into five primary types: cellular, tissue, organ, structure, and whole-body regeneration. Mitochondrial networks in each cell are highly dynamic in shape and subcellular distribution, which is often associated with their metabolic status. Although it is easier to observe the mitochondria in the restoration of damaged or missing cells, the dynamics and activity of mitochondria have been studied for all types of regeneration. Below, we summarize the discoveries in different model systems and discuss the molecular mechanisms underpinning animal regeneration (see illustration summary in Fig. 1).

### Cellular regeneration

Two model systems are widely used to study regeneration on the cellular level, neuron axons and nematode skin. *C. elegans* is an ideal platform for mitochondrial viewing *in* vivo due to its transparency and readily available fluorescent protein transgenic lines. In addition, the simple syncytial epithelium and highly extended morphology of neurons offer trackable systems for injury manipulations (Chisholm and Hsiao 2012; Chisholm and Xu 2012; Yanik et al. 2004). Mitochondria can actively respond to wounds in the syncytial epithelium and axons. In the syncytial epithelium, wounding triggers rapid and reversible mitochondrial fragmentation while increasing mtROS concentration, promoting actin polymerization and wound closure (Fu et al. 2020). In neurons, nerve injury results in the translocation of mitochondria to the injured axon, which is critical to the growth phase of regeneration (Han et al. 2016). In mice, injury to the sciatic nerve also induces the translocation and increased ER-mitochondrial tethering to the injured axon tip, which ultimately promotes axon regeneration (Lee et al. 2019; Zhou et al. 2016). The above studies also showed evidence that dysregulation of mitochondrial dynamics impaired the cellular regeneration processes, suggesting a conserved role of mitochondria in regulating cellular regeneration (Fu et al. 2020; Han et al. 2016; Lee et al. 2019; Zhou et al. 2016).

### Tissue and organ regeneration

Tissue and organ regeneration require the replenishment of missing cells. Skin, heart, and skeletal muscles are the three traditional model systems to study tissue and organ regeneration. Ultraviolet (UV) can induce mitochondrial fragmentation by translocation of a mitochondrial fission-promoting protein DRP1 to mitochondria in normal primary human keratinocytes (Juge et al. 2016). Mutations in mitochondrial proteins result in skin aging phenotypes, including the impairment of skin regeneration (Sreedhar et al. 2020). Cardiac regeneration was studied in zebrafish, axolotl, and mice. In the comparison of hearts capable of regeneration with those incapable by ^13^C-NMR isotopomer, lipidomic, and proteomic analyses, a clear area of difference has been identified to be a shift from anaerobic glycolysis to mitochondrial OXPHOS (Cardoso et al. 2020; Lopaschuk et al. 1992; Sakaguchi and Kimura 2021). Fatty acid-free milk from MMTV-Cre;*Perk*^*fl/fl*^ mice decreased cardiomyocyte proliferation of neonatal and adult mice. In contrast, knockout and pharmacological inhibition of *Pdk4* in cardiomyocytes promoted cardiomyocyte proliferation in adult mice, suggesting a viable therapeutic target for cardiac regeneration by inhibiting fatty acid oxidation and enhancing cardiac pyruvate dehydrogenase (PDH) activity (Cardoso et al. 2020; Sakaguchi and Kimura 2021). In skeletal muscle regeneration, the activation of resident quiescent stem cells (satellite cells) also requires fragmentation, a process that becomes more difficult in situations of inefficient OXPHOS and aging (Hong et al. 2022).

### Structure regeneration

The caudal fin of zebrafish and axolotl limb are popularly used systems in the study of structure regeneration. Unlike mammalian limbs, zebrafish, after fin injury, can form a blastema consisting of a layer of progenitor cells underneath the wounded epithelium. Increased ROS and wound-induced mitochondrial fragmentation have been detected in injured zebrafish tailfins (Fu et al. 2020; Romero et al. 2018). During adult axolotl limb regeneration, novel mitochondria-related musculoskeletal cell populations are revealed at the damaged region where the blastema is formed (Qin et al. 2021). Although it is hypothesized that mitochondria at the injured region offer enough energy and respiratory chain intermediates for stress reaction, cell differentiation, and tissue remodeling, the exact regulatory mechanisms remain unclear.

### Whole-body regeneration

Both hydra and flatworm planarians are typical animals investigated in studies of whole-body regeneration capacity (Elliott and Sánchez Alvarado 2013; Galliot 2012; Rink 2013). Inhibition of ROS by Diphenyleneiodonium (DPI) could block the regeneration of both hydra and planarian (Pirotte et al. 2015). In planarians, the adult stem cells, known as neoblasts, serve as the reservoir for regenerative cells (Reddien 2021). Previous studies have shown that planarian neoblasts contain low and high mitochondrial membrane potential (MMP) populations (Mohamed Haroon et al. 2021; Yang et al. 2020). A high throughput measurement of metabolism in planarians also revealed the activation of glycolysis during regeneration (Osuma et al. 2018). The function of mitochondrial metabolism in regulating planarian whole-body regeneration has to date not been demonstrated.

## Mitochondria-mediated molecular signaling for animal regeneration

Taking findings from prior studies in research organisms, transcription-independent and -dependent signals from mitochondria were characterized based on their requirement for regeneration. During regeneration, all cells adjacent to the surface of the injury can respond to the damage. In these processes, mitochondria play an important role depending on the metabolic homeostasis of aerobic glycolysis and the level of ROS, calcium, and ATP. Ca^2+^ and mtROS elevation together are considered the initiation of the response to injuries (Lansdown 2002; Raffaello et al. 2016; Stanisstreet 1982). Metabolic reprogramming and changes in mitochondrial networks follow this initial response to regulate downstream transcriptional cascade (Cardenas et al. 2010; Chandel 2015; Hong et al. 2022; Prieto et al. 2020; Sena and Chandel 2012). Mitochondria and mitoDAMPs can also be released from live or dying cells to be functional factors in sensing damage and regeneration (Brestoff et al. 2021; Liu et al. 2021; Niethammer 2016; Westhaver et al. 2022; Zhang et al. 2010).

### Mitochondrial ROS

ROS increase in response to injury and promote wound closure. Studies of *C. elegans* skin have demonstrated that mtROS superoxide increases after injury detected by the mtROS sensor cpYFP (Hou et al. 2012; Shen et al. 2014). Increasing the level of mtROS in *C. elegans* skin can promote wound repair as shown in SOD RNAi experiments (Xu and Chisholm 2014). This process, along with increased Ca^2+^ and treatment with pro-oxidant paraquat, results in a decrease of the actin ring diameter and promotion of wound closure (Xu and Chisholm 2014). Reduction of the ROS concentration by DPI or apocynin (APO) causes regeneration defects at wound sites of planaria, which have a vast capacity to regenerate their entire bodies (Pirotte et al. 2015).

In studies of axon damage, increased translocation of mitochondria to the nerve tip provides energy through the ETC required for regeneration. ROS, which are byproducts from the ETC, are useful second messenger signaling molecules that occupy an essential role in the wound healing and regeneration processes (Han et al. 2016; Lee et al. 2019).

ROS also plays a critical role in the proliferation of stem cells, which is considered the next phase in wound response. By transiently switching on in situ ROS production in mouse skin, Carrasco et al. demonstrated that ROS induced cell proliferation in tissue, promoted hair growth, and stimulated tissue repair after severe burn injury (Carrasco et al. 2015).

Inflammation is considered the initiating phase of the wound response. Surrounding the wound site, ROS act as the scavengers to promote wound closure correlated with upregulated M2-macrophage (Zhao et al. 2020). In skin-wound response, a subpopulation of early-stage wound macrophages is distinguished by mtROS production and HIF1α stabilization, both of which ultimately drive a pro-angiogenic program essential for timely healing (Willenborg et al. 2021). During injury, damaged cells can release endogenous mitochondria into circulation, the spread of which is capable of eliciting neutrophil-mediated wound healing (Zhang et al. 2010). This evidence suggests that mtROS and other mitochondrial products act as scavengers and serve a role in systematic immune response to promote the healing process.

Regulating mitochondrial ROS levels could result in improvements to regenerative processes. Treatment of CoQ10, which can decrease the ROS level in irradiated human skin fibroblasts, resulted in a decrease in mitochondrial dysfunction and preservation of skin health (Schniertshauer et al. 2016). Post-injury angiogenesis is widely considered a potential spinal cord injury (SCI) treatment strategy. The mitochondrial-specific antioxidant MitoQ, which is a ROS inhibitor, promotes functional recovery and tissue preservation through the enhancement of angiogenesis after SCI (Huang et al. 2022). Nasoohi et al. reported that CoQ10 supplementation could efficiently improve the functional deficit and cerebral infarction in stroke animals (Nasoohi et al. 2019; Ramezani et al. 2020). Therefore, the biological effects of mtROS may rely on different stages and cell types of animal regeneration.

### Metabolic reprogramming

Changes in mitochondrial metabolism regulate stem cell fate in many species. For example, mitochondria accumulate in the liver after injury to initiate the regeneration process. Thereafter, metabolic changes in mitochondria may induce different cell fates. Differentiated cells primarily rely on glucose metabolized by the tricarboxylic acid (TCA) cycle to generate the energy for cellular processes. In contrast, stem cells rely on glycolysis to provide energy regardless of oxygen availability. This phenomenon is termed “aerobic glycolysis”, and called the “Warburg effect”. Accumulating evidence indicates that the metabolic shift to glycolysis plays a vital role in regeneration. For instance, extracellular flux (XF) analysis indicated that metabolic changes occur with increased glycolysis during regeneration in planarians (Osuma et al. 2018). In Murphy Roths Large (MRL) mice, glycolysis has been demonstrated to be used during regeneration, and if enhanced, the OXPHOS in these mice led to inhibition of ear regeneration (Heber-Katz 2017). Some hypotheses show that a preference for aerobic glycolysis during regeneration in stem cells may result in a failure of the differentiation process (Ito and Ito 2016; Ito and Suda 2014).

Mitochondrial metabolic switches are related to cell fate in several systems (Ito and Ito 2016; Ludikhuize et al. 2020),. By LC–MS/MS, Rodriguez-Colman et al. showed that intestinal crypt-based columnar cells (CBC) adapt their respiration to maintain their stemness. Still, Paneth cells (PCs) adjacent to stem cells are mainly glycolytic in mice (Rodriguez-Colman et al. 2017). This indicates that metabolic rewiring of mitochondria during the differentiation of intestinal stem cells occurs. Transcriptomic data analysis suggested that glia cells could be reprogrammed to promote morphological and functional regeneration after CNS injury in *Drosophila* via increased glycolysis. This enhancement is mediated by the glia-derived metabolites L-lactate and L-2-hydroxyglutarate (L-2HG). Genetically/pharmacologically increasing or reducing the bioactivity of these metabolites promoted or impeded CNS axon regeneration (Li et al. 2020).

In aged mice, mitochondrial dysfunction occurs in quiescent muscle stem cells. However, stem cell function can be improved by NAD^+^ supplied through active mitochondrial unfolded protein response (UPR^mt^) which thereby increases mouse life span (Zhang et al. 2016). The NAD^ +^ -sirtuin pathway regulated longevity through UPR^mt,^ and the FOXO signal pathway and was identified by Laurent Mouchiroud et al. in *C. elegans* (Mouchiroud et al. 2013). A recent study suggested that Sirt1 regulates UPR^mt^ to promote tissue regeneration in zebrafish (Lin et al. 2021). Despite the classic M1/M2 phenotypes of macrophage, the functional phenotypic pattern of macrophage comes from the metabolic process regulated by mitochondria (Devanney et al. 2020). During wound response, efferocytosis for polarized macrophages was bolstered by apoptotic cell fatty acids, mitochondrial β-oxidation, the electron transport chain, and heightened coenzyme NAD^+^ (Zhang et al. 2019). Soluble advanced glycation end products (sRAGE) can serve as novel immunometabolic modulators that ameliorate ischemic stroke recovery by inhibiting the fatty acid synthesis and thus favoring CD4 + T cells polarization toward Treg after cerebral ischemia injury (Zhang et al. 2022). More importantly, Mao et al. demonstrated that mitochondrial abnormalities largely contributed to AGE-induced apoptosis of osteoblastic cells, as evidenced by enhanced mitochondrial oxidative stress, conspicuous reduction in mitochondrial membrane potential and adenosine triphosphate production, abnormal mitochondrial morphology, and altered mitochondrial dynamics (Mao et al. 2018).

### Mitochondrial morphological dynamics and activity

The metabolic transformation of mitochondrial dynamics occurs across cell types and statuses. Mitochondrial dynamics, including mitochondrial fusion and fission, are essential aspects of mitochondrial functioning and regulation of ROS, mitochondrial membrane potential, Ca^2+^, and homeostasis of cells. Mitochondrial fission promoted by FIS1 and DRP1 leads to fragmentation and forms granular mitochondria during mitosis and in response to acute injuries. Mitochondrial fusion promoted by OPA1 and MFN1/2 increases the length of mitochondria and forms tubular mitochondria, which is generally associated with cell differentiation. Lack of *Mfn* gene expression induces mitochondrial dysfunction and leads to defective development (Chen et al. 2010). In a recent study, mitochondrial dynamics were described as necessary for skeletal muscle satellite cells (SCs) to change their state from quiescence to proliferation. Upon knockdown of *Drp1* in injured tissue, SCs cannot functionally leave the quiescent state to proliferate and cause muscle regeneration (Hong et al. 2022). Moreover, acute brain injury and blood–brain barrier disruption could trigger the *Mfn2*-mediated formation of mitochondria-ER contact in astrocytes, which enables vascular remodeling (Gbel et al. 2020). In addition, mitochondrial dynamics is heterogeneous across different developmental stages. For example, in the mouse embryonic stage, neural stem cells have elongated mitochondria, but the shape of mitochondria is fragmented in adult bodies.

Differentiated cells with fragmented mitochondria decrease in mitochondrial mass. Mitochondrial dynamics downstream of FOXO/Notch signaling pathways in intestinal stem cells induce cell differentiation (Ludikhuize et al. 2020). Ludikhuize et al. demonstrated that the downregulation of FOXO induces stem cell differentiation into PCs. FOXO KD decreases respiration and induces mitochondrial fission through the upregulation of FIS1. Fission inhibited by Mdivi-1 treatment restores the increased fragmentation of mitochondria in the FOXO KD system. These results indicate that mitochondria are essential signaling organelles in stem cell maintenance and differentiation.

### Mitochondrial translocation

Transcellular transfer of mitochondria has emerged as a key example of cellular communication, which may be a mechanism of tissue regeneration. In previous studies, a conserved MAPK kinase, dual leucine zipper kinase (DLK), as a vital regulator in axonal damage signaling has been proven to be located at axons in response to damage in nematodes and mice (Hammarlund et al. 2009). Sung Min Han et al. found that the location of DLK is vital for mitochondrial translocation to the injured axon (Han et al. 2016). In Jung Eun Shin’s work, she demonstrated that DLK is necessary for retrograde mitochondrial transport of p-STAT3 injury signal in cells to promote regenerative programming (Shin et al. 2012). Unlike the peripheral nervous system (PNS), the central nervous system (CNS) lacks regenerative ability. Methods of CNS regeneration and the relationship between p-STAT3 and mitochondria translocation are in need of further study.

Intercellular transfer of mitochondria and the interaction of mitochondria with other cellular components also contribute to wound response and tissue regeneration. During injury, damaged cells can release endogenous mitochondria, the spread of which into the circulation could elicit a neutrophil-mediated wound response (Zhang et al. 2010). In recent studies, ginsenoside was shown to induce the transfer of astrocytic mitochondria to neurons and thereby act against ischemic stroke, which suggests a role for astrocytic mitochondria translocation in ischemic injury response (Ni et al. 2022). Intravitreal transplanted iPSC-MSCs can effectively donate functional mitochondria to retinal ganglion cells (RGCs) and protect against mitochondrial damage-induced loss of RGCs (Jiang et al. 2019). Lee et al. reported that increased ER-mitochondria tethering elevates mitochondrial Ca^2+^ and enhances ATP generation, thereby promoting the regrowth of injured axons (Lee et al. 2019). Torralba et al. uncovered that extracellular vesicles are transmitted from T cells to the dendritic cells containing genomic and mitochondrial DNA to trigger immune signaling in recipient cells (Torralba et al. 2018). These extracellular vesicles contain significant amounts of the transcription factor in addition to mitochondrial inner and outer membranes. Mild mitochondrial stress can protect mice from defects by enhancing UPR^mt^ activity. Mitochondria, as central hubs, provide energy for cellular activities and are also central to the stress response.

Cerebral ischemia–reperfusion (I/R) injury in the brain happens during the reperfusion of blocked blood due to the restoration of oxygen-rich blood. The main strategy to mitigate ischemic stroke injury is revascularization, which may lead to I/R injury (Phipps and Cronin 2020; Reis et al. 2017; Zerna et al. 2018). In a recent study, Xie et al. described that mitochondrial transplantation may attenuate cerebral I/R injury. This group demonstrated that mitochondrial transplantation could increase cell viability as well as decrease ROS and apoptosis levels. Mitochondrial transplantation provides a new therapeutic avenue for cerebral I/R injury. However, more studies are needed to uncover the mechanism of mitochondrial transplantation (Xie et al. 2021).

### Mitochondria-regulated signaling for stem cell proliferation and differentiation

All the above mechanisms represent transcriptional-independent factors. During animal regeneration, ROS, Ca^2^^ +^ , or metabolites induce the subsequent transcriptional response. In mouse skin, elevated ROS was associated with a transient Src kinase phosphorylation at Tyr416 and a strong transcription activation of prolactin family 2 subfamily c of growth factors (Carrasco et al. 2015). In *Drosophila*, mitochondrial ROS accumulation is essential for intestinal stem cell proliferation and lineage specification partially via repression of FOXO signaling (Zhang et al. 2020). ROS has also been shown to regulate the regeneration of zebrafish larval tails through relocation of Hedgehog expressing notochord cells to the wound site via an Src Family Kinase (SFK) signal pathway (Romero et al. 2018). Additionally, mutations in mtDNA can increase mitochondrial H_2_O_2_ production and decrease the reprogramming efficiency of mouse embryonic fibroblasts. This decreased reprogramming capacity can be rescued by the addition of N-Acetyl-L-cysteine (NAC) and mitochondrial ubiquinone (MitoQ) (Hamalainen et al. 2015).

During regeneration, the transition from the naïve to primed phase is companied by the stem cell from quiescence to proliferation state. Compared with naïve cells, primed human embryonic stem cells (hESCs) show an increased level of S-adenosyl-L-methionine (SAM), which is sustained in mitochondria as the methyl source of nicotinamide N-methyltransferase (NNMT) enzyme. Knockout of NNMT increased SAM levels and H3K27me3 marks and promoted the transition from naïve to primed stage (Sperber et al. 2015). How these signals from mitochondria regulate transcriptional and epigenetic changes will be the topics of further investigation.

## Conclusions and perspectives

UPR^mt^, protein translation and epigenetic modification are three major pathways to regulate downstream gene expression. Additionally, mitochondrial shape and dynamics can serve as essential inputs in determining cell fate and function (Chen and Chan 2017; Khacho and Slack 2018). Furthermore, mitochondrial crosstalk with the endoplasmic reticulum (ER) and lysosomes can regulate cell fate and function (Deus et al. 2020). The detailed molecular mechanisms are diverse across different cell types. The mitochondrial response may be a trigger for the cell cycle, which begins the differentiation program, or mitochondria may represent a unified center to promote cell fate. The question remains, how might mitochondria reprogram various transcription factors together with environmental factors? Cellular regeneration is easier to address in a homogenous, unchanging condition. The mechanism remains to be revealed in higher dimensional systems such as structure regeneration and whole-body regeneration.

As much evidence has shown mitochondrial direct or correlated function in animal regeneration, aging factors have also been noted to influence mitochondria-regulated animal regeneration. Mitochondria have been under extensive study in aging research. It is well-accepted that regeneration ability decreases in aged animals and the accumulation of mtDNA mutations has been demonstrated in tissue during aging and cultured conditions (Kujoth et al. 2007; Piko et al. 1988; Smith et al. 2022; Taylor and Turnbull 2005). The molecular mechanism of aging in animal regeneration is unknown and research is ongoing. Mitochondria, the center of metabolism, generate several key epigenetic modifiers regulating aging. Several studies have found that intervention strategies on mitochondrial metabolism such as OXPHOS and mitophagy could improve the regenerative ability of stem cells in aged tissues and organs. The function and potential tools to strengthen structures and whole-body regeneration are still unclear. Indeed, there may be conserved mechanisms across different types of animal regeneration. Utilization of the regulatory machinery present in biological systems may one day allow us to regenerate an entire arm or even an entire organism. Efforts in the future may need to pay attention to these fundamental considerations.

## Data Availability

Not applicable.

## Abbreviations

ATP: Adenosine triphosphate
mtDNA: Mitochondrial DNA
rRNAs: Ribosomal RNAs
tRNAs: Transfer RNAs
ROS: Reactive oxygen species
ER: Endoplasmic reticulum
OXPHOS: Oxidative phosphorylation
mitoDAMPs: Mitochondria-derived damage-associated molecular patterns
UV: Ultraviolet
PDH: Pyruvate dehydrogenase
DPI: Diphenyleneiodonium
MMP: Mitochondrial membrane potential
APO: Apocynin
ETC: Mitochondrial electron transport chain
CoQ10: Coenzyme Q10
SCI: Spinal cord injury
MitoQ: Mitoquinone
TCA: Tricarboxylic acid
XF: Extracellular flux
MRL: Murphy Roths Large
CBC: Crypt-based columnar cells
PCs: Paneth cells
CNS: Central nervous system
L-2HG: L-2-hydroxyglutarate
UPR^mt^: Mitochondrial unfolded protein response
NAD^+^: Nicotinamide adenine dinucleotide
sRAGE: Soluble advanced glycation end products
SCs: Satellite cells
FOXO: Forkhead box O
KD: Knock down
MAPK: Mitogen-activated protein kinase
DLK: Dual leucine zipper kinase
PNS: Peripheral nervous system
p-STAT3: Phosphorylated signal transducer and activator of transcription 3
RGCs: Retinal ganglion cells
I/R: Ischemia–reperfusion
SFK: Src Family Kinase
NAC: N-Acetyl-L-cysteine
hESCs: Human embryonic stem cells
SAM: S-adenosyl-L-methionine
NNMT: Nicotinamide N-methyltransferase
